# A Preclinical Rat Model of Heart Failure With Preserved Ejection Fraction With Multiple Comorbidities

**DOI:** 10.3389/fcvm.2021.809885

**Published:** 2022-01-13

**Authors:** Géraldine Hubesch, Aliénor Hanthazi, Angela Acheampong, Laura Chomette, Hélène Lasolle, Emeline Hupkens, Pascale Jespers, Grégory Vegh, Cécile Watu Malu Wembonyama, Caroline Verhoeven, Céline Dewachter, Jean-Luc Vachiery, Kathleen Mc Entee, Laurence Dewachter

**Affiliations:** ^1^Laboratory of Physiology and Pharmacology, Faculty of Medicine, Université Libre de Bruxelles, Brussels, Belgium; ^2^Department of Cardiology, Erasme Academic Hospital, Brussels, Belgium; ^3^Institut de Recherche Interdisciplinaire en Biologie Humaine et Moléculaire (IRIBHM), Université Libre de Bruxelles (ULB), Brussels, Belgium; ^4^Department of Mathematics Education, Faculty of Medicine, Université Libre de Bruxelles, Brussels, Belgium

**Keywords:** heart failure with preserved ejection fraction, diastolic dysfunction, metabolic syndrome, group 2 pulmonary hypertension, soluble ST2, RNA sequencing

## Abstract

Heart failure with preserved ejection fraction (HFpEF) is a common complex clinical syndrome for which there are currently few evidence-based therapies. As patients with HFpEF very often present with comorbidities comprising the metabolic syndrome, we hypothesized, that metabolic syndrome could lead over time to the development of diastolic dysfunction and HFpEF. Obesity-prone rats were exposed to high-fat diet and compared to obesity-resistant rats fed with standard chow. Phenotyping of metabolic syndrome, associated with echocardiographic and cardiac hemodynamic measurements, was performed after 4 and 12 months. Blood and myocardial tissue sampling were performed for pathobiological evaluation. High-fat diet in obesity-prone rats elicited metabolic syndrome, characterized by increased body and abdominal fat weights, glucose intolerance and hyperlipidemia, as well as increased left ventricular (LV) systolic pressure (after 12 months). This was associated with LV diastolic dysfunction (assessed by increased LV end-diastolic pressure) and pulmonary hypertension (assessed by increased right ventricular systolic pressure). Echocardiography revealed significant concentric LV hypertrophy, while LV ejection fraction was preserved. LV remodeling was associated with cardiomyocyte hypertrophy, as well as myocardial and perivascular fibrosis. Circulating levels of soluble ST2 (the interleukin-1 receptor-like) markedly increased in rats with HFpEF, while plasma NT-proBNP levels decreased. RNA-sequencing analysis identified clusters of genes implicated in fatty acid metabolism and calcium-dependent contraction as upregulated pathways in the myocardium of rats with HFpEF. High-fat diet during 12 months in obesity-prone rats led to the development of a relevant preclinical model of HFpEF with multiple comorbidities, suitable for investigating novel therapeutic interventions.

## Introduction

Heart failure with preserved ejection fraction (HFpEF) is a growing clinical and public health problem, accounting for approximately half of all cases of heart failure and associated hospital admissions (1). HFpEF is clinically defined as heart failure with normal ejection fraction and diastolic dysfunction, characterized by impaired relaxation and increased diastolic stiffness (2). All patients with HFpEF exhibit increased left ventricular (LV) filling pressure and reduced exercise tolerance (3, 4). However, pathophysiological mechanisms underlying disease progression of HFpEF are complex and remain poorly understood.

Although diastolic dysfunction is highly prevalent in HFpEF, the complex clinical phenotype that characterizes this syndrome stems from the presence of multiple cardiac and non-cardiac interrelated comorbidities, commonly seen in metabolic syndrome. These include obesity, systemic arterial hypertension, hyperlipidemia and *diabetes mellitus*, altogether contributing to the impairment of the cardiovascular reserve (5, 6). Furthermore, systemic inflammatory burden has been suggested to greatly contribute to HFpEF pathogenesis (7–9). HFpEF is more frequent in elderly patients (10, 11) and is closely associated with the presence of arterial hypertension as well as overweight or obesity (12). Moreover, up to 80% of patients with HFpEF develop pulmonary hypertension, which is associated with worse outcome and increased mortality (13).

HFpEF is a major unmet medical need and there is an urgent demand for new therapeutic approaches and strategies targeting mechanisms specific to HFpEF. However, definitive experimental evidence supporting these mechanisms has not emerged owing to limited success in developing a comprehensive preclinical model recapitulating the intricate pathophysiology of HFpEF and its global clinical picture. So far, evidence-based clinical therapies have failed to improve clinical symptoms, prognosis and mortality of HFpEF patients (8, 14, 15).

Because most HFpEF patients harbor multiple comorbidities, we propose a multiple-hit model created by inducing HFpEF in rats through metabolic stress (using a high-fat diet associated with a genetic predisposition) and prolonged follow-up timeline as a secondary stressor, to develop an animal model of HFpEF closest to human pathophysiology.

## Materials and Methods

### Animals and Blood and Tissue Sampling

All experimental procedures involving animals were approved by the Institutional Animal Care and Use Committee of the Faculty of Medicine of the *Université Libre de Bruxelles* (Brussels, Belgium; protocol acceptation number: 656N). Applicable guidelines were followed in accordance with the “Guide for Care and Use of Laboratory Animals” published by the US National Institutes of Health (NIH Publication eighth edition, update 2011).

Diet-induced obesity-prone (OP) and obesity-resistant (OR) Sprague-Dawley rats were raised using selected breeders obtained from Charles River Laboratories Inc. (Wilmington, MA, USA). These rats were used because their propensity to develop diet-induced obesity due to polygenic inheritance, thus closely mimicking the obesity in humans (16). Four-week-old OP (*n* = 10 and *n* = 14 in 4- and 12-month protocols respectively) and OR (*n* = 9 in both 4- and 12-month protocols) rats were enrolled in the study and kept under controlled environmental conditions (21°C, 60%-humidity atmosphere and 12-h light/dark cycles) along the protocol. OP rats were fed with a high-fat diet with 45% of energy from lipids and 17% from sucrose (473 kcal/100 g; D1245; Research Diets, New Brunswick, NJ, USA), whereas OR rats continued on standard rat chow (339 kcal/100 g with 5% of energy from lipids and 2% from sucrose; A03 Safe diets, Augy, France) for 4 and 12 months. Water and chow were given *ad libitum*. Body weight and food intake were monitored every week during the last 5 weeks of the protocols, to carefully characterize weight gain and energy ingestion. Food intake measured as food consumed per gram/day and calorimetry was evaluated.

**Figure 1:**
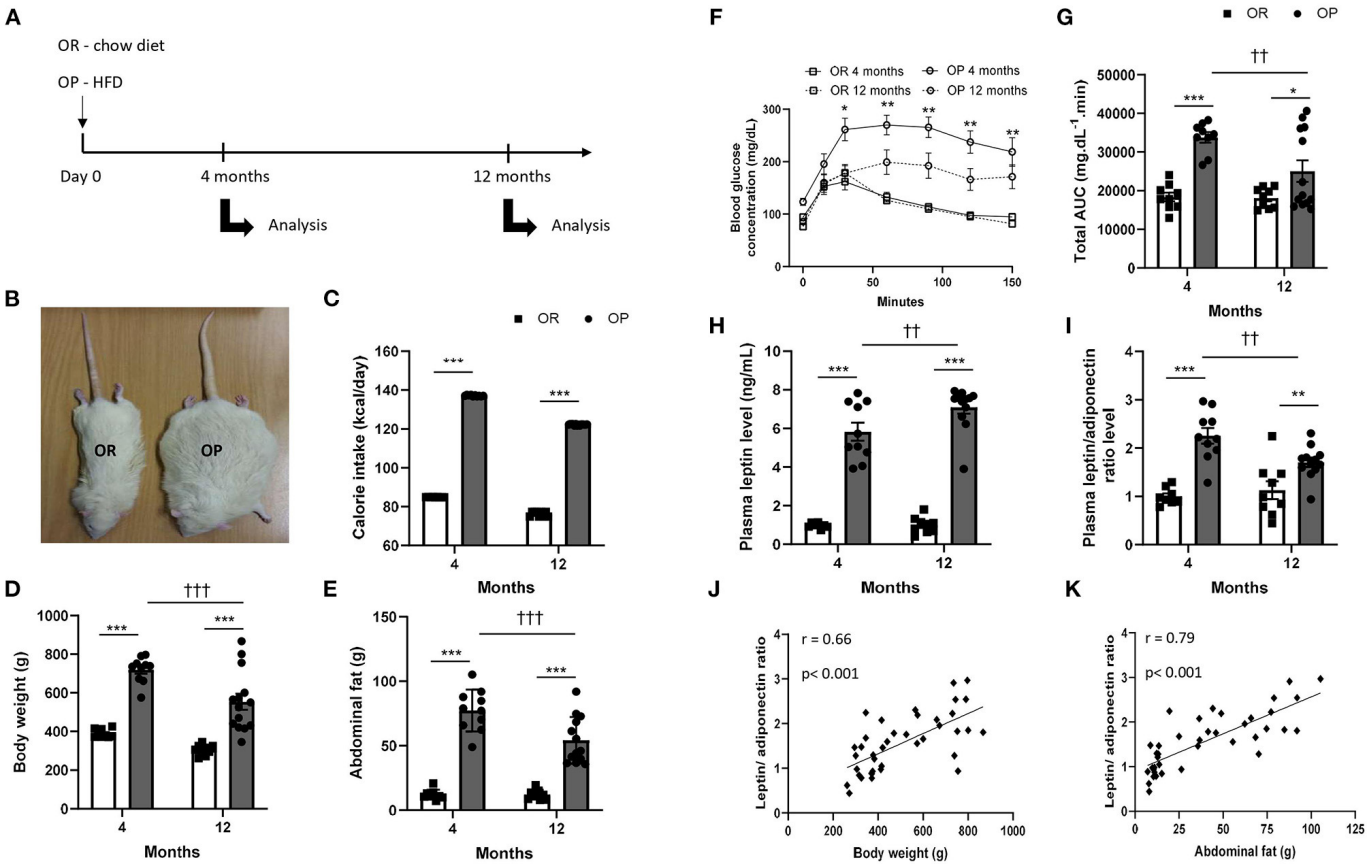
High-fat diet fed obesity-prone (OP) rats present all characteristics of metabolic syndrome after 12 months. **(A)** Experimental design. Obesity-resistant (OR) and obesity-prone (OP) rats were respectively maintained on normal standard chow or high-fat diet for 4 and 12 months and then analyzed. **(B)** Representative illustrative picture of high-fat diet fed obesity-prone (OP; on the right) vs. normal chow-fed obesity-resistant (OR; on the left) rats at 12 months. **(C)** Averaged daily calorie intake evaluated along the 4 and 12 months of the study, in high-fat diet-fed obesity-prone (OP; black bars) vs. normal chow-fed obesity-resistant rats (OR; white bars). Body **(D)** and total abdominal fat weights **(E)** of high-fat diet-fed obesity-prone (OP; black bars) vs. normal chow-fed obesity-resistant (OR; white bars) rats at 4 and 12 months. Glycemic response during fasting glucose tolerance test **(F)** and corresponding total area under the curve [AUC; (**G)**] in high-fat diet-fed obesity-prone (OP; black bars) vs. normal chow-fed obesity-resistant (OR; white bars) rats at 4 and 12 months. Data are presented as mean ± SEM (*n* = 8–14 rats per group). *0.01 < *p* < 0.05, **0.001 < *p* < 0.01, ***p < 0.001, high-fat diet in obesity-prone (OP) vs. normal chow in obesity-resistant (OR) rats; ^††^0.001 < *p* < 0.01, ^†*††*^*p* < 0.001, 4- vs. 12-month high-fat diet in obesity-prone (OP) vs. normal chow in obesity resistant (OR) rats. Plasma adipokine profile in high-fat diet-fed obesity-prone (OP) and normal chow-fed obesity-resistant (OR) rats after 4 and 12 months. **(H)** Plasma leptin levels and **(I)** leptin-to-adiponectin level ratio in high-fat diet-fed obesity-prone (OP; black bars) vs. normal chow-fed obesity-resistant (OR; white bars) rats at 4 and 12 months. Data are presented as mean ± SEM (*n* = 8–14 rats per group). ***p < 0.001, high-fat diet in obesity-prone (OP) vs. normal chow in obesity-resistant (OR) rats; ^††^0.001 < *p* < 0.01, 4- vs. 12-month high-fat diet in obesity-prone (OP) rats. Correlations between plasma leptin-to-adiponectin ratio and different characteristics of metabolic syndrome, including body weight **(J)** and total abdominal fat weight **(K)**. Data of all experimental groups and both 4- and 12-month protocol duration were gathered and analyzed together using a parametric Pearson's correlation coefficient analysis.

As illustrated in Figure 1A, at the end of the protocols (at 4- and 12-month time points), OP and OR rats were evaluated by echocardiography and invasive hemodynamic measurements. Before sacrifice, blood samples were drawn from the femoral vein, placed in EDTA-coated tubes and centrifuged (for 15 min at 1,500 g) to collect plasma for biochemical analyses and evaluation of adipokine and cardiac biomarker levels. Under deep anesthesia (isoflurane 5%), rats were finally euthanized by exsanguination (via section of the abdominal aorta). Adipose deposits from visceral, retroperitoneal, epididymal and mesenteric sites, and the hearts were rapidly harvested, weighted and processed for analysis. Hearts were snap-frozen in liquid nitrogen and stored at −80°C for RNA-sequencing (RNA-seq) analysis or, after a 24-h fixation in 10%-neutral buffered formalin, were embedded in paraffin for histopathological evaluations. Heart weights were normalized to tibial length to assess cardiac hypertrophy independently of body weight.

### Biochemical Analysis

To analyze potential alterations in glucose metabolism, a glucose-tolerance test was performed by intraperitoneal injection of glucose (2 g.kg^−1^ in saline) after overnight fasting. Glucose levels (mg.dL^−1^) were measured, with a portable glucometer (Contour XT kit, Bayer HealthCare, Loos France), from a drop of blood taken at the tip of the tail before (0 min) and at 15, 30, 60, 90, 120 and 150 min after glucose administration. Glucose tolerance was obtained from the area under the curve (obtained from the serum glucose concentration *vs*. time points), using Graphpad Prism 9.0 (Graphpad software, San Diego, California, USA).

Concentrations of triglycerides, total cholesterol and HDL-cholesterol were determined using Cobas8000® in plasma samples, according to manufacturer's instructions. Plasma concentration of LDL-cholesterol was calculated.

### Plasma Levels of Adipokines and Cardiac Biomarkers

Plasma concentrations of leptin and adiponectin were determined with rat leptin (KRC2281; Thermo Fisher Scientific, Waltham, MA, USA) and adiponectin (RRP300; R&D Systems, Minneapolis, USA) enzyme-linked immunosorbent assay (ELISA) kits respectively, according to manufacturers' instructions. Results were presented as the mean value of duplicated experiments.

Plasma levels of cardiac biomarkers, including N-terminal (NT) pro-B-type natriuretic peptide (BNP) and interleukin-1 receptor-like 1 (IL-1RL1, commonly called sST2), were evaluated using rat NT-proBNP (MBS012301; MyBioSource, San Diego, USA) and sST2 (MBS9348955; MyBioSource, San Diego, USA) ELISA kits respectively, according to manufacturers' instructions. Results were presented as the mean value of duplicated experiments.

### Echocardiography

Transthoracic 2D, M-mode and Doppler echocardiography was performed using a digital ultrasound machine (Vivid-7, GE Healthcare, Etat, USA) equipped with a 12-MHz phased array transducer (Hewlett Packard, Palo Alto, CA) to assess cardiac structure and function. Animals were sedated with an intraperitoneal injection of ketamine (24 mg.kg^−1^) and medetomidine (0.32 mg.kg^−1^) and placed in right and left lateral recumbent positions on a heating pad to control body temperature. Sedation was confirmed by lack of response to firm pressure on one of the hind paws. Electrocardiogram was monitored via limb leads throughout the procedure. All measurements were obtained by the same observer according to methods recommended by the American Society of Echocardiography currently applied to humans (17).

Two-dimensional M-mode and pulsed-wave Doppler echocardiography was performed in the right parasternal (long- and short- axis) and subcostal views. Diastolic (d) and systolic (s), septal (SWT) and posterior wall thickness (PWT) and LV diameters (LVID) were measured in M-mode from a LV short axis view at the level of *chordae tendinae* and fractional shortening (FS) was calculated. LV ejection fraction (LVEF) was derived using the Teichholz formula. To estimate LV hypertrophy, relative wall thickness ((SWTd + PWTd)/LVEDD), relative wall area (LV epicardial short-axis area—LV endocardial short-axis)/LV epicardial short-axis area) were calculated. LV mass was calculated using the American Society of Echocardiography recommended formula: LV Mass = 1.05 (5 /6 × A × L), where A (epicardial area—endocardial area) is planimetered short-axis area obtained at the papillary muscle level, and L is the LV length (apex to mid-mitral annulus plane) obtained from the parasternal long-axis view normalized for tibial length, to assess LV weight independently of body weight. Aortic flow was measured from the subcostal window to calculate forward stroke volume and cardiac output. All parameters were measured for at least three heart beats, at end-diastole and end-systole. An averaged value for each animal was used.

### Hemodynamic Measurements

Invasive hemodynamic evaluation through close-chest LV and right ventricular catheterization was performed in spontaneously breathing rats under anesthesia using a rodent anesthesia system (Minerve, Esternay, France; isoflurane: 4% for induction, 2% during surgery and 1% while performing hemodynamic measurements). Left carotid artery was cannulated with a microtip pressure catheter (rodent catheter 1.6F, Transonic, The Netherlands), which was gently placed in the middle of the left ventricle. LV end-systolic and end-diastolic pressures were measured. LV end-systolic pressure was used for systemic vascular resistance calculation. Thereafter, the microtip pressure catheter was moved and placed in the middle of the right ventricle through the right external jugular vein cannulation. Correct anatomic placement was confirmed by respective pressure contours. After, right ventricular systolic pressure was measured. Hemodynamic data were recorded and analyzed with a ADV500 PV data acquisition system (Transonic, AD Instruments, The Netherlands) after stabilizing the pressure line for each animal.

### Histological Analysis—Cardiac Morphometry

Three-micrometer myocardial sections were taken along the transversal axis of the heart and stained with hematoxylin-eosin for overall morphological analysis, as previously described (18). Briefly, mean cross-sectional areas of cardiomyocytes were calculated by measuring at least 50 cells for each myocardial sample (at a 400-fold magnification in light microscopy). From these hematoxylin-eosin-stained myocardial sections, structural vascular density (number of cardiac vessels/mm^2^) was evaluated using at least 20 randomly selected microscopic fields (at a 200-fold magnification in light microscopy) of myocardial sections. All morphological analyses were performed using a LEICA DFC425C camera and LEICA DM2000 microscope (Leica Microsystems; Heerbrugg, Germany) and Image J analysis software (imagej.nih.gov/ij/) in a blind fashion by two independent investigators. Averaged values of cross-sectional areas of myocardial cells and of number of cardiac vessels were used as indicators of cardiomyocyte size and myocardial vascular density respectively.

Myocardial interstitial and perivascular fibrosis were evaluated by Masson's Trichrome and by Picrosirius red staining to assess the presence of collagen accumulation and fibrosis within myocardial sections. Picrosirius red staining was also observed under polarized light microscopy.

### Quantification of Nitric Oxide (NO) Metabolite (Nitrite) Production

Four mm-length segments of freshly collected thoracic aorta were incubated during one hour with Krebs solution-Henseleit solution (118 mmol.L^−1^ NaCl; 4.7 mmol.L^−1^ KCl; 1.2 mmol.L^−1^ MgSO_4_; 1.2 mmol.L^−1^ KH_2_PO_4_; 2.5 mmol.L^−1^ CaCl_2_; 25 mmol.L^−1^ NaHCO_3_; 5.1 mmol.L^−1^ glucose; Merck, Darmstadt, Germany) in a 24-well cell culture plate. Supernatants were collected and the levels of NO were measured indirectly by the determination the nitrite levels, using the Measure-iT^TM^ High-Sensitivity Nitrite Assay kit (Molecular Probes, Eugene, USA) according to the manufacturer's instructions. Briefly, collected supernatants were ultra-filtered through a 10 000-molecular weight cut-off filter to eliminate proteins (VWR; Leuven, Belgium). Fluorescence was measured in triplicate with a microplate reader at 365/450 nm. Nitrite concentrations were obtained by referring to a standard curve realized in parallel and expressed in mol.L^−1^.

### RNA Extraction and RNA-Seq Data Acquisition

Total RNA was extracted from three randomly chosen snap-frozen myocardial tissue samples from 12-month OP and OR rats, using TRIzol reagent (Invitrogen, Merelbeke, Belgium), followed by a chloroform/ethanol extraction and a final purification using QIAGEN RNeasy® Mini kit (QIAGEN, Hilden, Germany), according to manufacturer's instructions. RNA sample concentration was determined with a standard spectrophotometer Nanodrop® (ND-1000; Isogen Life Sciences, De Meern, The Netherlands) and RNA integrity was assessed by visual inspection of GelRed (Biotium, Hayward, California)-stained agarose gels. RNA sample quality was finally checked using a Fragment Analyzer 5200 (Agilent, Santa Clara, CA, USA).

RNA-seq was performed at the Brussels Interuniversity Genomics High Throughput core (www.brightcore.be). Briefly, indexed cDNA libraries were obtained using the TruSeq RNA sample preparation kit (Illumina, San Diego, CA, USA) following the manufacturers' instructions. The multiplexed libraries were loaded on a Novaseq 6000 (Illumina) using a S2 flow cell and sequences were produced using a 200 Cycle Kit. Approximately 25-million paired-end reads per sample were mapped against the *rattus norvegicus* (Rnor 6.0) reference genome using STAR software (version STAR_2.5.3a) to generate read alignments for each sample. Annotations Rnor 6.0.103.gtf were obtained from ftp.Ensembl.org. After transcripts assembling, gene level counts were obtained using HTSeq tool (version HTSeq_0.9.1).

### Gene Ontology and iDEP Pathway Analysis of Differentially Expressed Genes

Hierarchical clustering and differentially expressed genes between high-fat diet fed OP rat and standard chow fed OR rat group were obtained using the integrated website application for analysis of RNA-seq data iDEP (http://bioinformatics.sdstate.edu/idep/). The genes with a *p*-value < 0.05 and an absolute log-fold change > 1 were selected for further evaluation. The volcano plot was constructed using VolcaNoseR (https://huygens.science.uva.nl/VolcaNoseR/). Gene ontology (GO) and pathways enrichment analysis was applied to these differentially expressed genes using Enrichr (Mouse Pathways database) (http://amp.pharm.mssm.edu/Enrichr). In parallel, manual literature analysis was realized for each differentially expressed gene regarding their molecular function in the rats and implication in HFpEF. Heatmap visualization was realized using R software v4.0.3 (https://www.r-project.org). The corresponding detailed results of RNA sequencing analysis are available at the GEO number: GSE189190.

### Real-Time Quantitative Polymerase Chain Reaction (RTq-PCR)

Reverse transcription was performed using random hexamer primers and Superscript II Reverse Transcriptase (Invitrogen, Merelbeke, Belgium), according to the manufacturer's instructions.

For RTq-PCR, sense and antisense primers were designed using Primer3 program for *rattus norvegicus* superoxide dismutase (SOD) 1 and 2, glyceraldehyde 3-phosphate dehydrogenase (GAPDH) and hypoxanthine phosphoribosyl transferase (HPRT) 1 mRNA sequences. To avoid inappropriate amplification of residual genomic DNA, intron-spanning primers were selected when exon sequences were known. For each sample, amplification reaction was performed in triplicate using SYBR Green PCR Master Mix (Quanta Biosciences, Gaithersburg, MD, USA), specific primers and diluted template cDNA. Result analysis was performed using an iCycler System (BioRad Laboratories). Relative quantification was achieved by normalization with the housekeeping genes, GAPDH and HPRT1.

### Statistical Analysis

All data were presented as mean ± standard error of the mean (SEM). Statistical analysis was performed using Graphpad Prism 9.0 (Graphpad, San Diego, California, USA). Intergroup comparisons were tested by two-factor ANOVA for multiple measurements and LVd mass results were tested with one-way ANOVA. When the F ratio of these analyzes reached a critical *p*-value < 0.05, comparisons were made with a parametric Student *t*-test followed by Bonferroni correction as *post-hoc* test. A value of *p* < 0.05 was considered as statistically significant; n represents the number of individual data.

Correlations were analyzed parametrically by the determination of the Pearson correlation coefficient and non-parametrically by the Spearman's rank correlation coefficient.

## Results

### High-Fat Diet in Obesity-Prone Rats Leads to Metabolic Syndrome

#### General and Abdominal Obesity

At baseline, body weights of 4-week-old OP and OR rats were similar (93 ± 2 *vs*. 93 ± 1 g in OP *vs*. OR rats). During the last 5 weeks of 4- and 12-month protocols, food intake was recorded and corresponding daily calorie intake was calculated and averaged. Despite their high-calorie diet (45% kilocalories from lipids and 17% from sugars), OP rats showed a higher daily food consumption (Figure 1C). Total daily calorie intake was greater by 35% in OP rats eating a high-fat diet compared to standard chow-fed OR rats (Figure 1C) independently of protocol length. Consumption of a high-fat diet for 4 and 12 months in OP rats promoted higher body (Figures 1B,D) and abdominal fat (Figure 1E) weight gains. These markers of general and intra-abdominal (visceral) obesity were already present and even higher after 4 compared to 12 months (85- *vs*. 82%-increases in body weight and 541- *vs*. 348%-increases in abdominal fat weight after 4 *vs*. 12 months respectively, in OP compared to OR rats; Figures 1D,E).

#### Metabolic Profile

Plasma levels of triglycerides, total cholesterol, and low- (LDL) and high-density (HDL)-cholesterol, were increased after 4 months, and even more so after 12 months of high-fat diet in OP rats (Table 1), suggesting aggravated dyslipidemia over time. The ratio of LDL-to-HDL-cholesterol was 3- and 4-fold increased after 4- and 12-month high-fat diet respectively in OP rats compared to their lean counterparts (Table 1).

**Table 1:** Evolution of plasma lipid profile in high-fat diet-fed obesity-prone and in normal chow-fed obesity-resistant rats during 4 and 12 months.

	**4 months**			**12 months**			***p* 4- vs. 12-month high fat diet in OP rats**
	**Normal chow in OR rats (*n* = 8)**	**High fat diet** **in OP rats** **(*n* = 10)**	***p***	**Normal chow** **in OR rats ** **(*n* = 8)**	**High fat diet in OP rats (*n* = 14)**	***p***	
Triglycerides (in mg.dL^−1^)	90 ± 9	218 ± 27	***	131 ± 16	910 ± 136	***	^†*††*^
Total cholesterol (in mg.dL^−1^)	55 ± 2	109 ± 9	*	73 ± 4	214 ± 22	***	^†*††*^
HDL-cholesterol (in mg.dL^−1^)	44 ± 1	66 ± 5	**	54 ± 2	86 ± 6	***	^††^
LDL-cholesterol (in mg.dL^−1^)	10 ± 1	43 ± 7	***	19 ± 2	128 ± 19	***	^†*††*^
LDL/HDL ratio	0.23 ± 0.02	0.69 ± 0.14	*	0.35 ± 0.03	1.55 ± 0.20	***	^†*††*^

Values are expressed as means ± SEM. *0.01 < p < 0.05, **0.001 < p < 0.01, ***p < 0.001, high-fat diet-fed in obesity-prone (OP) vs. normal chow-fed in obesity-resistant (OR) rats. ^††^0.001 < p < 0.01, ^†††^p < 0.001, 12- vs. 4-month high-fat diet-fed in obesity-prone (OP) rats. HDL means high-density lipoprotein; LDL, low-density lipoprotein; OR, obesity-resistant rats; OP, obesity-prone rats.

As illustrated in Figure 1F, fasting blood glucose levels were similar in OP rats fed with a 4- and 12-month high-fat diet compared to OR rats. In order to test the effects of high-fat diet on metabolic parameters in OP rats, we used an intraperitoneal glucose tolerance test to assess glucose sensitivity. Serum glucose levels at 30, 60, 90, 120 and 150 min were significantly higher in OP rats fed with high-fat diet during 4 months than those observed in standard chow-fed OR rats with an apparent lower clearance (Figure 1F). Serum glucose curves did not return to their basal glucose levels 150 min after glucose administration in both 4- and 12-month high-fat diet-fed OP rat groups. Total AUC of serum glucose levels between 0 and 150 min were shown for the four groups of rats in Figure 1G. The AUC of 4- and 12-month high-fat diet-fed OP rats were higher than those observed in corresponding OR rats, with maximal AUC-value after 4 months of high-fat dieting. This suggested that high-fat diet in OP rats induced glucose intolerance at 4 and 12 months and that this was more pronounced at 4 months.

Because adipokines play important roles in the pathophysiological link between dysfunctional adipose tissue and cardiometabolic alterations, we evaluated the effects of high-fat diet on plasma adipokine levels. Plasma concentration of leptin was increased in OP rats after a 4-month and much more after a 12-month high-fat diet (Figure 1H). The concentration of adiponectin was also increased in these animals (4.2 ± 0.6 *vs*. 1.8 ± 0.4 μg.mL^−1^ and 7.5 ± 0.9 *vs*. 1.6 ± 0.4 μg.mL^−1^ in high-fat diet-fed OP compared to OR rats after 4 and 12 months respectively; *p* < 0.001). Leptin-to-adiponectin ratio, which has been proposed as a marker of adipose tissue dysfunction (19), was increased by 2-fold in high-fat diet-fed OP rats after 4 months (Figure 1I). After 12 months, this ratio was slightly lower compared to 4 months, but remained higher in obese rats compared to their lean counterparts (Figure 1I). This leptin-to-adiponectin ratio was correlated to body (Figure 1J) and abdominal fat (Figure 1K) weights.

#### Systemic Hypertension

**Figure 2:**
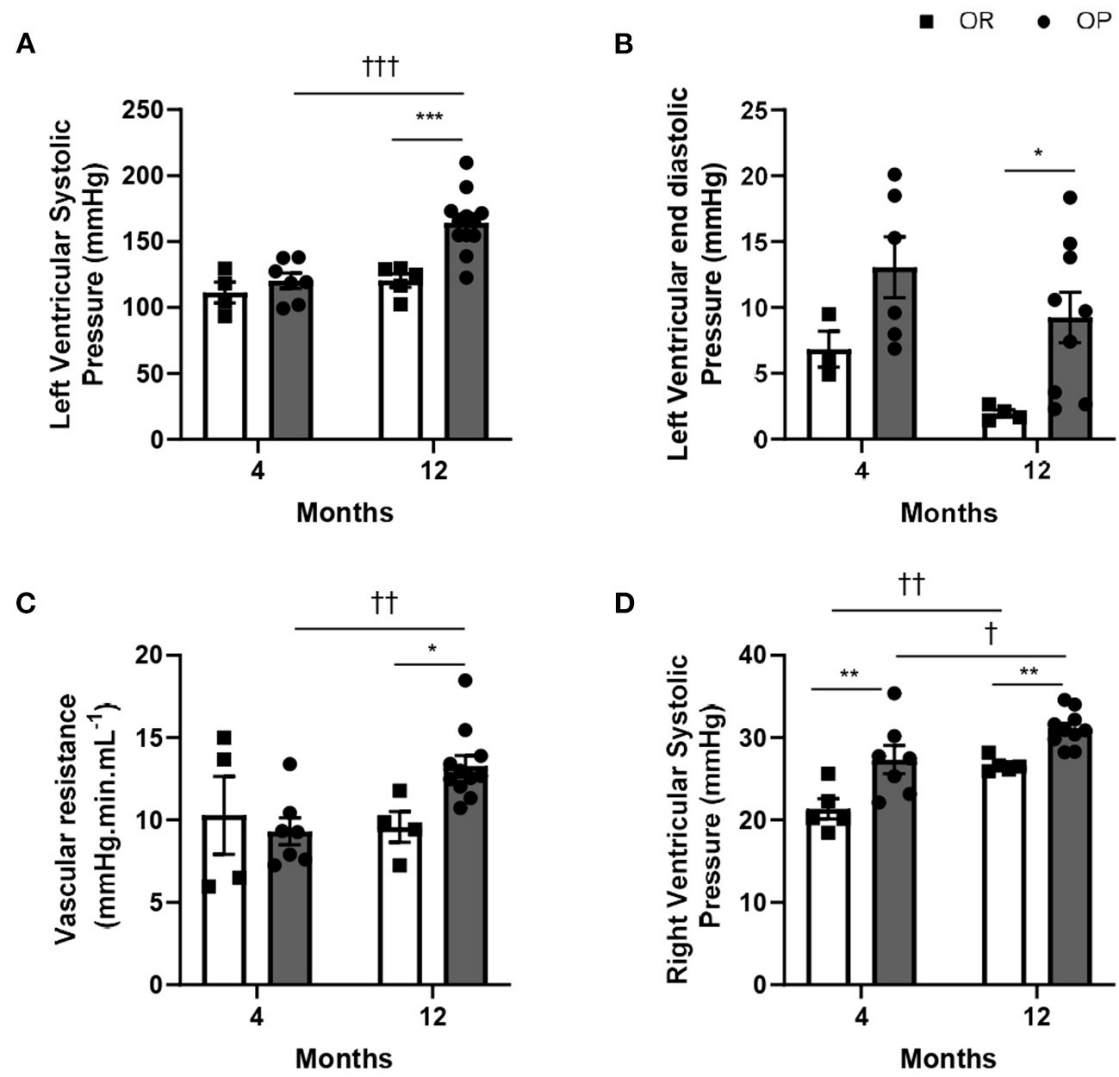
Hemodynamic characterization of high-fat diet-fed obesity-prone (OP) and normal chow-fed obesity-resistant (OR) rats after 4 and 12 months. **(A)** Left ventricular systolic (LVSP) and **(B)** end-diastolic pressures in high-fat diet-fed obesity-prone (OP; black bars) vs. normal chow-fed obesity-resistant (OR; white bars) rats at 4 and 12 months. **(C)** Total systemic vascular resistance was calculated as the ratio between the left ventricular systolic pressure (evaluated by closed-chest left ventricular catheterization) and the normalized cardiac output (evaluated by echocardiography) in high-fat diet-fed obesity-prone (OP; black bars) vs. normal chow-fed obesity-resistant (OR; white bars) rats at 4 and 12 months. **(D)** Right ventricular systolic pressure in high-fat diet-fed obesity-prone (OP; black bars) vs. normal chow-fed obesity-resistant rats (OR; white bars) at 4 and 12 months. Data are presented as mean ± SEM (*n* = 8–14 rats per group). *0.01 < *p* < 0.05, **0.001 < *p* < 0.01, ***0.001 < *p*, high-fat diet in obesity-prone (OP) vs. normal chow in obesity-resistant (OR) rats; ^†^0.01 < *p* < 0.05, ^††^0.001 < *p* < 0.01, ^†*††*^*p* < 0.001, 4- vs. 12-month high-fat diet in obesity-prone (OP) or obesity-resistant (OR) rats.

While LV systolic pressure was similar in both groups of rats after 4 months, high-fat diet was associated with an increase in LV systolic pressure after 12 months in OP rats fed with high-fat diet (Figure 2A).

#### Characterization of Metabolic Syndrome

All characteristics of metabolic syndrome, except alterations in LV pressures, were already present after 4-month high-fat diet in OP rats. Even if the phenotype of metabolic syndrome and associated metabolic characteristics were less exacerbated (than after 4 months), LV systolic pressure was markedly increased after 12-month protocol (Figure 2A), showing LV pressure overload.

### Metabolic Syndrome Evolves to HFpEF Overtime

#### Hemodynamic Evaluation

High-fat diet in OP rats was associated with increased LV end-diastolic pressure after 12 months, while no change was observed after 4 months (Figure 2B). As illustrated in Figure 2C, high-fat diet in OP rats increased systemic vascular resistance by 24% after 12 months. Right ventricular catheterization using a closed-chest approach was performed to evaluate pulmonary pressure in these rats. After 4 and 12 months, metabolic syndrome resulted in the development of mild pulmonary hypertension (assessed by increased right ventricular systolic pressure) in OP rats fed with high-fat diet (Figure 2D).

#### LV Structure

**Figure 3:**
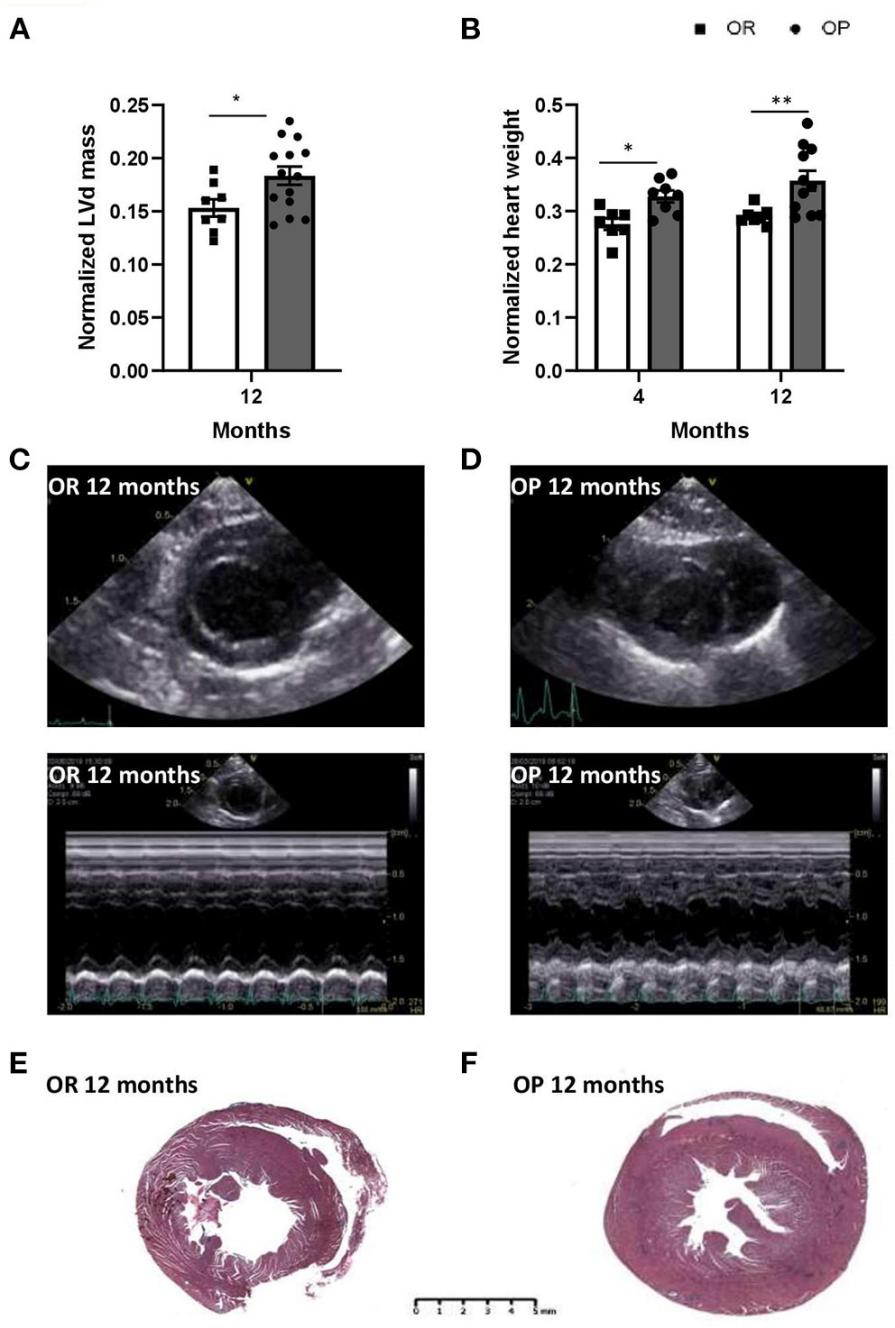
High-fat diet-fed obesity-prone (OP) rats display, after 12 months, key alterations found in clinical HFpEF. **(A)** Echocardiographic evaluation of left ventricle mass normalized to tibial length (in mm) in high-fat diet fed obesity-prone (OP; black bars) vs. normal chow fed obesity-resistant (OR; white bars) rats at 12 months. **(B)** Ratio of heart weight (in g) to tibial length (TL; in cm) in high-fat diet-fed obesity-prone (OP; black bars) vs. normal chow-fed obesity-resistant rats (OR; white bars) at 4 and 12 months. Data are presented as mean ± SEM (*n* = 8–14 rats per group). *0.01 < *p* < 0.05, **0.001 < *p* < 0.01, high-fat diet in obesity-prone (OP) vs. normal chow in obesity-resistant (OR) rats. Representative left ventricular bi-dimensional (top) and M-mode echocardiographic (bottom) tracings in normal chow fed obesity-resistant [OR; **(C)**] and high-fat diet-fed obesity-prone [OP; **(D)**] rats at 12 months. Images are representative of 10 independent rats. Representative Masson Trichrome-stained myocardial macro cross-sections at ventricle level from normal chow-fed obesity-resistant [OR; **(E)**] and high-fat diet-fed obesity-prone [OP; **(F)**] rats at 12 months. Scale bar: 5 mm.

**Figure 4:**
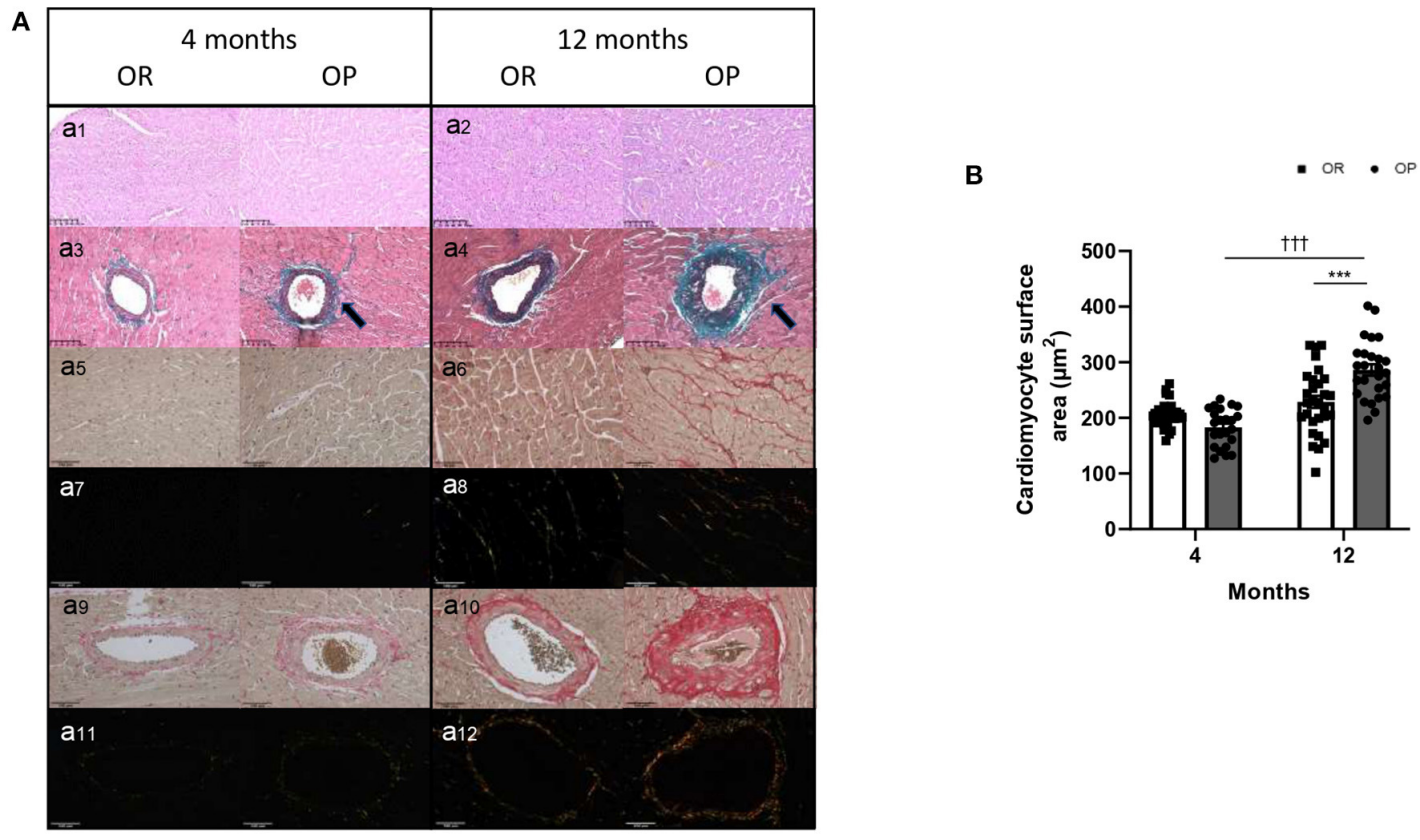
Myocardial structure, including cardiomyocyte hypertrophy and fibrosis, in high-fat diet-fed obesity-prone (OP) and normal chow-fed obesity-resistant (OR) rats after 4 and 12 months. **(A)** Representative images of hematoxylin and eosin-stained sections of left ventricle (a1,2) from high-fat diet-fed obesity-prone (OP) and normal chow-fed obesity-resistant rats (OR) at 4 (a1) and 12 months (a2). Representative Masson Trichrome-stained sections of myocardial vessels (a3,4) from high-fat diet-fed obesity-prone (OP) and normal chow-fed obesity-resistant rats (OR) at 4 (a3) and 12 months (a4). Trichrome Masson staining was performed to detect fibrotic areas [collagen fibers stained in green; indicated by arrows in a3,4]. Representative images of Picrosirius red-stained sections of left ventricle observed using classical and polarized light microscopy (a5–8 respectively) and of myocardial vessels (a9–12 respectively) from high-fat diet-fed obesity-prone (OP) and normal chow-fed obesity-resistant rats (OR) at 4 and 12 months. Myocardial interstitial and vascular sections were obtained at 200-fold magnification. Scale bars: 100 μm. **(B)** Cardiomyocyte hypertrophy (assessed by cardiomyocyte area in square micrometer) in left ventricles from high-fat diet-fed obesity-prone (OP; black bars) and normal chow-fed obesity-resistant rats (OR; white bars) at 4 and 12 months. Data are presented as mean ± SEM (25 images for each animal; *n* = 2–3 rats per group). ***0.001 < *p*, high-fat diet in obesity-prone (OP) vs. normal chow in obesity-resistant (OR) rats; ^†*††*^*p* < 0.001, 4- vs. 12-month high-fat diet in obesity-prone (OP) rats.

Echocardiography was performed in the two groups of rats after 4 and 12 months to assess cardiac structure and function. As illustrated in Table 2, there was no difference in heart rates between groups at both 4- and 12-month time points. All echocardiographic parameters measured after 4 months were similar between OP rats fed with high-fat diet and their lean counterparts (Table 2). After 12 months, metabolic syndrome resulted in significant LV wall hypertrophy (Figure 3), as evaluated by increased LV mass (standardized for tibial length; Figure 3A), relative wall thickness (RWT) and relative wall area (Table 2; Figures 3C,D). This concentric LV hypertrophy assessed by echocardiography was confirmed, at autopsy, by whole heart (Figure 3B) and cardiomyocyte (Figure 4) morphometric analysis. Total heart weight (normalized to tibial length) was similarly increased after 4 and 12 months in OP rats fed with high-fat diet (Figure 3B). The LV of the 12-month high-fat diet-fed OP rats presented obvious cardiomyocyte hypertrophy, characterized by an increase in cell surface area compared to obesity-resistant rats (Figures 4Aa1,2,B). In contrast, after 4-month high-fat diet, cardiomyocyte surface area was reduced compared to OR rats (Figure 4B). Because myocardial interstitial fibrosis and collagen deposition influence the mechanical properties of the myocardium, we used Masson's Trichrome (Figure 4Aa3,4) and Picrosirius red (Figure 4Aa5–12) staining to detect fibrotic area. LV fibrosis, characterized by interstitial and perivascular fibrotic deposition, was exacerbated in high-fat diet-fed OP rats after 12 months (Figure 4Aa5–12). Capillary density, determined as the number of capillaries/μm^2^ of myocardial tissue, was evaluated in LV cross sections. No differences were observed between the four groups of rats (19 ± 3 *vs*. 19 ± 2 and 16 ± 2 *vs*. 16 ± 1 capillaries/mm^2^ myocardial tissue section in OP *vs*. OR rats after 4 and 12 months respectively, p> 0.05 for all comparisons). Consistently, capillary-to-cardiomyocyte ratio remained unchanged between groups (data not shown). Myocardial infiltration with inflammatory cells (performed on hematoxylin-eosin-stained sections) was similar in all 4 groups of rats (data not shown).

**Table 2:** Two-dimensional and M-mode echocardiographic parameters of left ventricular structure and function in high-fat diet-fed obesity-prone and in normal chow-fed obesity-resistant rats during 4 and 12 months.

	**4 months**			**12 months**			***p* 4- vs. 12-month high fat diet in OP rats**
	**Normal chow in OR rats (*n* = 8)**	**High fat diet in OP rats** **(*n* = 10)**	***p***	**Normal chow** **in OR rats (*n* = 8)**	**High fat diet in OP rats (*n* = 14)**	***p***	
Heart rate (in beats/min)	258 ± 13	257 ± 10	NS	227 ± 5	208 ± 8	NS	NS
Relative LV wall surface area	0.53 ± 0.01	0.52 ± 0.02	NS	0.54 ± 0.01	0.59 ± 0.02	*	^††^
LVEF (in %)	63 ± 2	68 ± 5	NS	63 ± 2	69 ± 3	NS	NS
FS (in %)	30 ± 1	34 ± 3	NS	30 ± 1	35 ± 3	NS	NS
LVIDd (in mm)	1.80 ± 0.04	1.77 ± 0.07	NS	1.78 ± 0.05	1.79 ± 0.08	NS	NS
LVRWT	0.42 ± 0.02	0.47 ± 0.03	NS	0.42 ± 0.02	0.51 ± 0.03	*	NS
Stroke volume (in mL)	0.26 ± 0.04	0.25 ± 0.02	NS	0.23 ± 0.01	0.28 ± 0.02	0.09	NS
Cardiac output (in mL/min)	15.3 ± 3.4	14.0 ± 1.3	NS	13.6 ± 0.4	13.2 ± 0.8	NS	NS

Values are represented as means ± SEM. ^*^0.01 < p < 0.05, high-fat diet fed in obesity-prone (OP) vs. normal chow fed in obesity-resistant (OR) rats. ^††^0.001 < p < 0.01 12- vs. 4-month high-fat diet fed in obesity-prone (OP) rats. Relative left ventricular wall surface area was calculated as follows: (LVAd sax EPI – LVAd sax PM)/(LVAd sax EPI), where LVAd sax EPI means left ventricular epicardial short-axis area at the papillary muscle tip level at end-diastole, and LVAd sax PM, left ventricular endocardial short-axis at the papillary muscle tip level at end diastole and was used to estimate left ventricular hypertrophy. Stroke volume was calculated as follows: (LVOT VTI x aortic cross-sectional area), where LVOT VTI means left ventricular outflow tract velocity time integral (used as a measure of cardiac systolic function and cardiac output) and the aortic cross-sectional area (in mm). Cardiac output was calculated with the formula (stroke volume x heart rate). LV means left ventricular; LVEF, left ventricular ejection fraction; FS, fractional shortening; LVIDd, left ventricular internal diastolic dimension (indexed for tibia length); LVRWT, relative wall thickness; OR, obesity-resistant rats; OP, obesity-prone rats; NS, not significant.

#### LV Systolic Function

After 4 and 12 months, LV cavity sizes remained within the normal range and were similar in OP and OR rats, as assessed by unchanged LVIDd adjusted for tibial length. LVEF remained unaltered, with normal values above 60% (Table 2). No significant differences in FS (with normal values above 25%), stroke volume and cardiac output were observed (Table 2). Altogether, this strongly suggested preserved LV systolic function in OP rats with metabolic syndrome induced by a 12-month high-fat diet.

#### Cardiac and Endothelial Biomarkers

**Figure 5:**
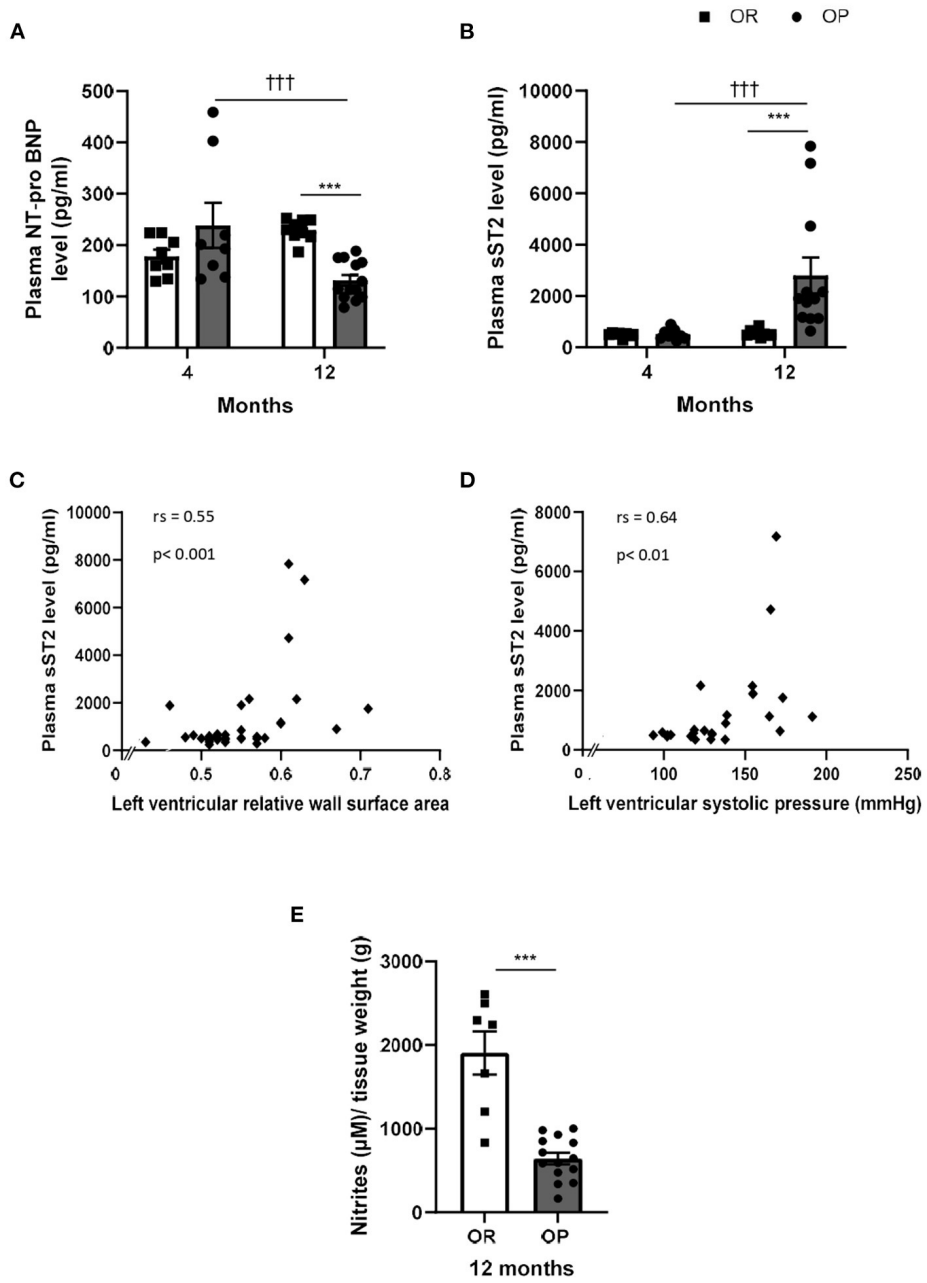
Plasma cardiac biomarkers in high-fat diet-fed obesity-prone (OP) and normal chow-fed obesity-resistant (OR) rats after 4 and 12 months. Plasma levels of N-Terminal pro-B-type natriuretic peptide [NT-proBNP; **(A)**] and soluble suppression of tumorigenicity 2 [sST2; **(B)**] in high-fat diet-fed obesity-prone (OP; black bars) vs. normal chow-fed obesity-resistant rats (OR; white bars) at 4 and 12 months. Data are presented as mean ± SEM (*n* = 8–14 rats per group). ***p < 0.001, high-fat diet in obesity-prone (OP) vs. normal chow in obesity-resistant (OR) rats; ^†*††*^*p* < 0.001 4- vs. 12-month high-fat diet in obesity-prone (OP) rats. Correlations between plasma soluble ST2 levels and left ventricular structure and hemodynamic parameters, including left ventricular relative surface area **(C)** and left ventricular systolic pressure [LVSP; **(D)**] respectively. Data of all experimental groups and both 4- and 12-month protocol duration were gathered and analyzed together using a non-parametric Spearman's rank correlation coefficient analysis. Concentration of nitrites/NO **(E)** in supernatants of endothelium-intact thoracic aortic rings collected in 12-month high-fat diet-fed obesity-prone rats (OP; black bars) vs. normal chow-fed obesity resistant rats (OR; white bars) incubated during one hour with Krebs solution. Data are presented as mean ± SEM (*n* = 9–14 rats per group). ***p < 0.001 high-fat diet in obesity-prone (OP) vs. normal chow in obesity-resistant (OR) rats.

Circulating levels of cardiac diagnostic biomarkers for heart failure and cardiac dysfunction, including NT-proBNP and sST2, were evaluated in the two groups of rats at both time points. After 4 months, plasma levels of NT-proBNP were not significantly different between OP and OR rats, while NT-proBNP levels decreased after 12 months in rats with metabolic syndrome compared to their lean counterparts (Figure 5A). Plasma sST2 levels markedly increased in OP rats fed with high-fat diet during 12 months, while sST2 remained low after 4 months (Figure 5B). Plasma sST2 levels were correlated to LV relative wall area (Figure 5C) and LV systolic pressure (Figure 5D). As illustrated in Figure 5E, release of NO was decreased in thoracic aorta collected in 12-month high-fat diet fed OP rats compared to controls.

#### Pathobiological Myocardial Characterization

**Figure 6:**
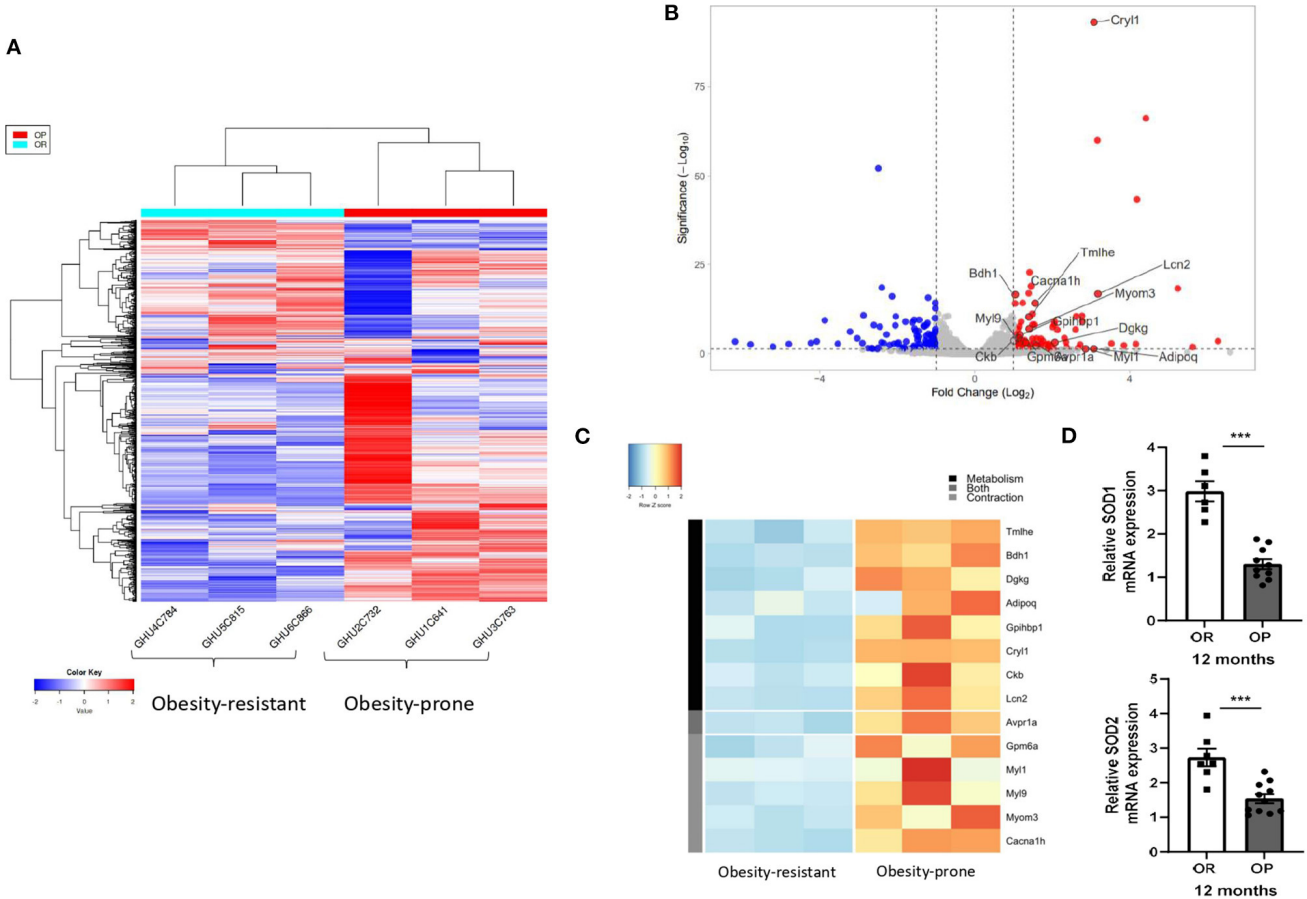
Myocardial RNA sequencing (RNA-seq) data analysis identified clusters of genes implicated in fatty acid metabolism and calcium-dependent contraction in high-fat diet-fed obesity-prone (OP) after 12 months. Heat-map **(A)** showing hierarchical clustering of genes from RNA-seq in three randomly chosen normal chow-fed obesity-resistant rats (OR; on the left) and in three randomly chosen high-fat diet-fed obesity-prone rats (OP; on the right) at 12 months. Raw Z score are shown on the heatmap. Volcano plot **(B)** of RNA-seq transcriptome data displaying the pattern of gene expression values. Y-axis denotes – log10 *p*-values, while X-axis shows log2 fold change values. Volcano plot was generated using VolcaNoseR. Two clusters of differently expressed genes implicated in fatty acid metabolism and calcium-dependent contraction are represented **(C)** and full gene lists are available in Supplementary Table 1. **(D)** Relative mRNA expression of superoxide dismutase 1 (SOD1) and 2 (SOD2) in myocardial tissue samples from normal chow-fed obesity-resistant rats (OR; white bar) and from high-fat diet-fed obesity-prone rats (OP; black bar) at 12 months. Data are presented as mean ± SEM (*n* = 6–11 rats per group). ***p < 0.001 high-fat diet in obesity-prone (OP) vs. normal chow in obesity-resistant (OR) rats.

The RNA-seq analysis identified 165 differentially expressed genes between the 2 groups of rats after 12-month protocol (Figure 6A; Supplementary Table 1 for detailed results). Analysis indicated that many of the genes that were upregulated in OP rats fed with high-fat diet during 12 months were implicated in energy substrate use (Figures 6B,C). Genes implicated in fatty acid synthesis, such as trimethyllysine hydroxylase-ε (TMLHE) and diacylglycerol kinase-γ (DGKG), and catabolism (3-hydroxybutyrate dehydrogenase-1; BDH1) were upregulated, as well as other molecules implicated in fatty acid transport, such as glycosylphosphatidylinositol anchored high-density lipoprotein binding protein 1 (GPIHBP1) and lipocalin 2 (Lcn2) (Figure 6B). Creatine Kinase B (CKB), an enzyme implicated in energy homeostasis, was also upregulated (Figures 6B,C). As illustrated Figure 6B, the RNA-seq analysis also identified an upregulated cluster of genes implicated in calcium-dependent contraction, including glycoprotein (GPM6A), myosin light chain 1 (MYL1) and 9 (MYL9) and myomesin 3 (MYOM3), as well as a calcium voltage-gated channel subunit-α (CACNA1H) in 12-month high-fat diet fed OP rats. Arginine vasopressin receptor 1A (Avpr1a), a receptor implicated in both glycogenolysis and contraction was also upregulated in 12-month high-fat diet OP rats (Figures 6B,C). Myocardial mRNA expression of SOD1 and SOD2, two antioxidant enzymes were decreased in OP rats fed with high-fat diet during 12 months (Figure 6D).

#### Characterization of HFpEF

OP rats fed with high-fat diet during 12 months presented with elevated LV end-diastolic pressure (Figure 2B), global myocardial hypertrophy (Figure 3) and preserved LVEF (Table 2), with increased right ventricular systolic pressure (Figure 2D), which strongly suggested the development of HFpEF appearing secondarily to metabolic syndrome.

## Discussion

Given the limitations of current preclinical models, we developed a rat model of HFpEF that recapitulates the vast majority of the clinical features of the syndrome. Metabolic syndrome induced by prolonged high-fat feeding during 12 months in OP rats, leads to HFpEF characterized by increased LV filling pressures and diastolic dysfunction, adverse structural and functional LV remodeling (with cardiomyocyte hypertrophy and fibrosis), and preserved ejection fraction, together with elevated right ventricular systolic pressure. This was associated with increased circulating levels of sST2, while NT-proBNP levels remained low. At pathobiological level, myocardial RNA-sequencing analysis identified clusters of genes implicated in fatty acid metabolism and calcium-dependent contraction, as the most disrupted pathways in rats with HFpEF.

In patients, HFpEF is characterized by signs and symptoms of heart failure with evidence of cardiac structural and functional abnormalities consistent with the presence of LV diastolic dysfunction, in the presence of a normal LV ejection fraction (20). Although systolic function was preserved in the present experimental model, there was an increase in LV diastolic pressure. This is consistent with the pivotal criteria in establishing a diagnosis of HFpEF which remains in the evidence of LV filing pressure elevation, indicative of a diastolic dysfunction or impaired ability of the heart to fill during diastole, in presence of a non-dilated LV and a preserved LV ejection fraction (in the absence of significant mitral regurgitation) (21, 22). However, one limitation of the present study is that it was not possible to perform traditional measures of LV diastolic dysfunction in rats, including E/A, E/e' and Doppler flow velocities. Although the precise mechanism leading to myocardial stiffening remain undetermined, LV stiffness is thought to have active and passive components. Cardiac remodeling, including myocardial hypertrophy, inflammation and fibrosis, have been shown to play crucial roles in the pathophysiology of HFpEF, all leading to an impaired LV relaxation ability (23, 24). In the present study, LV remodeling, characterized by significant concentric LV hypertrophy and myocardial fibrosis (both interstitial and perivascular), was found in high-fat diet-fed OP rats, ultimately leading to a loss of LV compliance and increased LV filling pressures (25).

Even if we found diastolic dysfunction (assessed by increased LV end-diastolic pressure) and significant alterations in LV structure, together with preserved LVEF, assessment of signs and symptoms of heart failure remain difficult (or even impossible) in rodents, as already discussed (26, 27). They are known to interfere with normal animal behavior and exercise capacity, although these may not be specific to HFpEF. In the present study, we did not evaluate exercise capacity *per se*, but we noticed that high-fat diet fed OP rats with HFpEF were more sedentary and moved less in their cages than their lean counterparts. Recently, two diagnostic algorithms, the HFA-PEFF (28)and the and H_2_FPEF scores (11) used as novel clinical standards for defining the key clinical features of HFpEF, have been transposed to experimental models of HFpEF (26). Here, we found a HFA-PEFF score of 4 (corresponding to diastolic dysfunction and alteration in LV morphological aspects without increased natriuretic peptide levels) and a H_2_FPEF score of 5 (related to the presence of obesity, hypertension, pulmonary hypertension and diastolic dysfunction) corresponding to an intermediate and a high score for HFpEF respectively. These 2 scores validated and confirmed the translational value of the present experimental model of HFpEF.

Obesity and associated metabolic risks have been proposed as a major driver of LV diastolic dysfunction and HFpEF, contributing to systemic inflammation and subsequent myocardial remodeling (7, 29, 30). Obesity has direct and indirect effects on the heart, including increased myocardial load associated with plasma volume expansion, worsening of systemic arterial hypertension, LV hypertrophy and increased aortic stiffness (31). In the present study, a worsening of the cardiovascular disease was observed with increasing age in high-fat diet-fed OP rats, even if their body weight was not increasing. HFpEF is also recognized to be an age-related disease (11). Heart failure is indeed disproportionately distributed among elderly individuals, as over half of all patients hospitalized with heart failure are older than 75 years, with 50% presenting with diastolic dysfunction (32). The present experimental model, obtained in genetically obesity-predisposed rats on a 12-month high-fat diet, recapitulates key comorbidities observed in patients with HFpEF, such as obesity, early metabolic derangements and pressure overload. After 12 months, OP rats on high-fat diet developed signs of HFpEF, characterized by elevated LV end-diastolic pressure and LV hypertrophy, while LV ejection fraction was preserved. LV systolic function remained normal, whereas diastolic function was impaired, which strongly suggests an early-stage of HFpEF after 12 months.

Systemic hypertension is one of the main underlying conditions, leading to HFpEF (33, 34). Hypertension, which causes broad changes in inflammation and metabolism, can cause myocardial stiffness and diastolic dysfunction (35). Insulin resistance may also be a key factor underlying the link between obesity and HFpEF (36), as the central driver of systemic microvascular inflammation and subsequent myocardial dysfunction (7). In the present study, LV pressure overload and glucose intolerance were present, when diastolic dysfunction and HFpEF were observed. Obesity is known to amplify comorbidities, such as insulin resistance and hypertension, partly through the adipose tissue which is capable of releasing regulatory factors, such as adipokines involved in promoting a global systemic pro-inflammatory state. Consistently, the risk of all-cause mortality was significantly higher in patients with HFpEF with abdominal obesity than in those without abdominal obesity (37). Here, we found increased abdominal fat weight, together with increased levels of adiponectin and leptin, with high leptin-to-adiponectin ratio in OP rats on high-fat diet. This is consistent with previous data showing elevated levels of leptin and adiponectin in HFpEF (38). The impact of obesity on HFpEF pathophysiology encompasses hemodynamic, neurohumoral and inflammatory mechanisms, altogether contributing to the reduction of the normal relaxation ability of the LV as the ventricular wall becomes stiffer from increasing interstitial fibrosis.

Utilization of relevant biomarkers is of clinical interest, because HFpEF is often difficult to diagnose early. Natriuretic peptides, which are produced under cardiac pressure/volume overload and reflects the degree of myocardial stretching and dysfunction (39), represent the current gold standard of biomarkers for the diagnosis, management and prognosis of heart failure (40). In patients with HFpEF, natriuretic peptides are mainly elevated in patients with advanced diastolic dysfunction, but are frequently in the normal range in mild diastolic dysfunction (41). Extra-cardiac factors, such as age, obesity and renal function are known to influence its measurement. Moreover, natriuretic peptide testing has yielded false negative results in 20% of obese patients with heart failure (42), with a risk of a false-negative BNP value around 20%, and for NT-proBNP about 15% (43). In the present study, NT-proBNP levels remained low, probably due to the presence of obesity (also observed in humans), while circulating levels of sST2 were largely increased in animals with HFpEF. This is consistent with previous reports showing elevated circulating inflammatory and reduced cardiac stretch biomarkers in HFpEF (44–47). We did not evaluate the inflammatory biomarker level of C-reactive protein (CRP), but characterized level of sST2, a marker of inflammation and fibrosis, which has been suggested to play a crucial role in ventricular remodeling and fibrosis in the context of pressure overload (48). In patients with HFpEF, sST2 has been linked to LV functional impairment assessed by global longitudinal strain (49) and associated with patient outcome (50). sST2 may represent the recently proposed concept that a systemic pro-inflammatory state driven by coexisting conditions is the additional cause of myocardial remodeling and dysfunction leading to HFpEF (7, 8). Here, we found that serum sST2 levels were correlated with LV overload and hypertrophy, suggesting that elevated sST2 may reflect LV alterations in HFpEF. In this context, circulating sST2 levels could provide additional diagnostic value beyond NT-proBNP levels for detecting LV diastolic dysfunction and early HFpEF, especially in obese patients.

Epidemiological studies have shown that pulmonary hypertension due to LV diastolic dysfunction, also referred to as pulmonary hypertension associated to HFpEF, is the most prevalent form of pulmonary hypertension associated to left heart disease (51, 52). Pulmonary hypertension is closely associated with worse outcome and mortality in patients with HFpEF. In HFpEF, chronically elevated LV filling pressures cause a passive backward transmission of pressures in the pulmonary arteries, resulting in increased pulmonary arterial pressure and pulmonary vascular resistance (53). In addition, pulmonary hypertension exacerbates the LV diastolic dysfunction already occurring in the heart (54). Pulmonary artery pressure increased also with aging (55). The present experimental model recapitulates key clinical features known to be present in HFpEF patients who develop PH (26). The pulmonary artery pressure observed in 12-month high-fat diet fed OP rats was in the range of what is observed in HFpEF patients developing pulmonary hypertension (right ventricular systolic pressure around 46–51 mmHg) (52, 54).

In OP rats fed with high-fat diet during 12 months, exaggerated interstitial and perivascular fibrosis and cardiomyocyte hypertrophy were observed, while myocardial infiltration with inflammatory cells and capillary density remained unchanged. This cardiac hypertrophic phenotype was associated, at molecular level, with the upregulation of genes implicated in fatty acid use and in calcium-dependent contraction. These two gene clusters have both been implicated in mechanisms tempting cardiac adaptation to various stressors (24). Intriguingly, no inflammatory or cell survival markers were found. This strongly suggested that cardiac molecular signature in HFpEF reflects a myocardial response to extra-cardiac comorbidities, such as obesity, dyslipidemia, insulin resistance or hypertension, that may contribute to cardiomyocyte hypertrophy and altered contraction, which seem to ultimately concurring to the reduced compliance of the myocardium (56). A paradigm for HFpEF development was proposed, which identifies a systemic proinflammatory state induced by comorbidities as the cause of myocardial structural and functional alterations. Indeed, this seems to induce myocardial microvascular endothelial inflammation and oxidative stress, which both contribute to reduced bioavailability of NO and decreased protein kinase G activity in cardiomyocytes. Resulting hypertrophy and stiffness in cardiomyocytes together with myocardial interstitial fibrosis played important roles in the development of diastolic dysfunction and HFpEF (7). Here, we found decreased expression of SOD1 and 2, which are first line antioxidant enzymes of defense against reactive oxygen species (ROS). Myocardial alteration in ROS elimination in HFpEF rats could probably contribute to myocardial oxidative stress. In HFpEF rats, we also showed decreased thoracic aorta release of NO, strongly suggesting endothelial dysfunction in these rats. However, the link between oxidative stress and endothelial dysfunction should be further investigated in next studies to understand better the pathogenesis of diastolic dysfunction in metabolic syndrome-associated HFpEF.

Given the heterogeneity in the HFpEF syndrome, any animal model of HFpEF only resembles a certain proportion of the patients. In the present study, we chose to develop an experimental model of HFpEF associated to obesity. Previous studies have already described obesity-based experimental models of HFpEF in rodents (26, 27, 57), but most of them presented discrepancies compared to the human phenotype. Preclinical obesity models usually present with rapid uncontrolled hyperglycemia and insulin resistance secondary to type 2 diabetes (58, 59), which does not mimic the clinical situation of HFpEF (60, 61). In our experimental model of HFpEF associated to obesity, we found glucose intolerance (but no type 2 diabetes) with low fasting glucose levels, which was close to the human condition. Additionally, obesity was mostly experimentally induced with specific genetic manipulations targeting the leptin signaling (58, 59, 62, 63). Because they do no exhibit similar abnormalities as do obese HFpEF patients, these experimental models are less pathophysiologically and clinically relevant. The originality of the present experimental model is that obesity naturally evolved with increasing age to HFpEF, mimicking the natural clinical overtime evolution of HFpEF in obese patients.

The present study validated a high-fat diet-induced rat model of early HFpEF developing pulmonary hypertension, which may offer a new avenue for testing potential mechanisms and therapeutic interventions, such treatment with the promising sodium-glucose co-transporter 2 (SGLT2) inhibitors that have shown recently beneficial effects in patients with HFpEF (64).

## Data Availability Statement

The datasets presented in this study can be found in online repositories. The names of the repository/repositories and accession number(s) can be found below: www.ncbi.nlm.nih.gov/GSE189190.

## Ethics Statement

The animal study was reviewed and approved by Institutional Animal Care and Use Committee of the Faculty of Medicine of the Université Libre de Bruxelles (Brussels, Belgium; protocol acceptation number: 656N).

## Author Contributions

GH, LD, and KME conceived and designed study. GH, AH, AA, EH, PJ, GV, CW, and KME performed research. GH, LC, HL, PJ, CV, CD, and KME analyzed data. GH, LC, CD, J-LV, KME, and LD contributed new methods or models. GH and LD wrote the paper. All authors have given approval to the final version of the manuscript.

## Funding

This work was supported by funds from the Fonds pour la Chirurgie Cardiaque (Brussels, Belgium).

## Conflict of Interest

The authors declare that the research was conducted in the absence of any commercial or financial relationships that could be construed as a potential conflict of interest.

## Publisher's Note

All claims expressed in this article are solely those of the authors and do not necessarily represent those of their affiliated organizations, or those of the publisher, the editors and the reviewers. Any product that may be evaluated in this article, or claim that may be made by its manufacturer, is not guaranteed or endorsed by the publisher.
