# Supplementary material for: A Preclinical Rat Model of Heart Failure With Preserved Ejection Fraction With Multiple Comorbidities
Source: Front Cardiovasc Med. 2022 Jan 13;8:809885. doi: 10.3389/fcvm.2021.809885 (PMC8793630; doi:10.3389/fcvm.2021.809885)
Supplement: Supplementary file 1 [file Table_1.DOCX]

Supplementary Material

# Supplementary Table – Gene list

| **Symbol** | **Gene name** | **Log2 FC** | p-value |
| --- | --- | --- | --- |
| H3f3c | H3 Histone Family Member 3C | 4.42 | 6.7E-67 |
| Fcer2 | Fc Fragment Of IgE Receptor II | 4.19 | 3.6E-44 |
| Fam83c | Family With Sequence Similarity 83 Member C | 4.16 | 1.9E-03 |
| Chga | Chromogranin A | 3.53 | 1.4E-03 |
| Lcn2 | Lipocalin 2 | 3.18 | 1.8E-17 |
| Magt1 | Magnesium Transporter 1 | 3.17 | 1.1E-60 |
| Cryl1 | Crystallin Lambda 1 | 3.08 | 8.3E-94 |
| Adipoq | Adiponectin, C1Q And Collagen Domain Containing | 3.07 | 4.5E-02 |
| Myl1 | Myosin Light Chain 1 | 2.86 | 4.5E-02 |
| Mpz | Myelin Protein Zero | 2.77 | 2.5E-11 |
| Tgm3 | Transglutaminase 3 | 2.72 | 2.8E-03 |
| Lrrc9 | Leucine Rich Repeat Containing 9 | 2.67 | 2.7E-02 |
| Slc17a7 | Solute Carrier Family 17 Member 7 | 2.62 | 3.4E-11 |
| Pik3c2g | Phosphatidylinositol-4-Phosphate 3-Kinase Catalytic Subunit Type 2 Gamma | 2.60 | 1.7E-07 |
| Adtrp | Androgen Dependent TFPI Regulating Protein | 2.37 | 1.5E-02 |
| Efcab6 | EF-Hand Calcium Binding Domain 6 | 2.35 | 5.9E-04 |
| Atp6ap1l | ATPase H+ Transporting Accessory Protein 1 Like | 2.32 | 4.3E-05 |
| Avil | Advillin | 2.13 | 2.0E-07 |
| Dgkg | Diacylglycerol Kinase Gamma | 2.07 | 6.9E-04 |
| Pcp2 | Purkinje Cell Protein 2 | 2.06 | 1.0E-09 |
| Tpsb2 | Tryptase Beta 2 | 2.05 | 5.0E-09 |
| Wdfy4 | WDFY Family Member 4 | 1.98 | 5.0E-03 |
| Gfap | Glial Fibrillary Acidic Protein | 1.88 | 2.1E-02 |
| Scube3 | Signal Peptide, CUB Domain And EGF Like Domain Containing 3 | 1.88 | 3.1E-02 |
| Gldn | Gliomedin | 1.85 | 2.9E-03 |
| Sfrp5 | Secreted Frizzled Related Protein 5 | 1.76 | 2.8E-04 |
| Chl1 | Cell Adhesion Molecule L1 Like | 1.71 | 1.5E-03 |
| Tpsab1 | Tryptase Alpha/Beta 1 | 1.71 | 6.4E-05 |
| Cadm4 | Cell Adhesion Molecule 4 | 1.69 | 1.2E-02 |
| Sypl2 | Synaptophysin Like 2 | 1.63 | 3.2E-03 |
| Sox10 | SRY-Box Transcription Factor 10 | 1.60 | 5.1E-05 |
| Avpr1a | Arginine Vasopressin Receptor 1A | 1.58 | 4.9E-03 |
| Cacna1h | Calcium Voltage-Gated Channel Subunit Alpha1 H | 1.56 | 7.3E-15 |
| Dnah7 | Dynein Axonemal Heavy Chain 7 | 1.54 | 7.8E-09 |
| Adam23 | ADAM Metallopeptidase Domain 23 | 1.52 | 1.1E-03 |
| Sncg | Synuclein Gamma | 1.49 | 7.2E-09 |
| Chrdl1 | Chordin Like 1 | 1.49 | 7.1E-05 |
| Ecd | Ecdysoneless Cell Cycle Regulator | 1.49 | 7.3E-12 |
| Ifi27 | Interferon Alpha Inducible Protein 27 | 1.45 | 1.2E-19 |
| Sostdc1 | Sclerostin Domain Containing 1 | 1.44 | 3.6E-03 |
| Rpl30 | Ribosomal Protein L30 | 1.41 | 1.6E-23 |
| Myom3 | Myomesin 3 | 1.41 | 1.1E-07 |
| Tmlhe | Trimethyllysine Hydroxylase, Epsilon | 1.40 | 5.0E-11 |
| Ddah2 | Dimethylarginine Dimethylaminohydrolase 2 | 1.39 | 1.3E-17 |
| Anxa8 | Annexin A8 | 1.38 | 4.8E-02 |
| Tmem163 | Transmembrane Protein 163 | 1.36 | 4.5E-02 |
| Rps4y2 | Ribosomal Protein S4 Y-Linked 2 | 1.34 | 3.0E-03 |
| Gpm6a | Glycoprotein M6A | 1.32 | 7.8E-04 |
| E2f7 | E2F Transcription Factor 7 | 1.31 | 2.1E-03 |
| Col28a1 | Collagen Type XXVIII Alpha 1 Chain | 1.31 | 2.4E-02 |
| Cdh26 | Cadherin 26 | 1.30 | 3.4E-02 |
| Gpr37l1 | G Protein-Coupled Receptor 37 Like 1 | 1.29 | 1.8E-04 |
| Cdh19 | Cadherin 19 | 1.28 | 1.1E-03 |
| Card19 | Caspase Recruitment Domain Family Member 19 | 1.24 | 6.0E-15 |
| Plekhb1 | Pleckstrin Homology Domain Containing B1 | 1.22 | 1.5E-04 |
| Ecrg4 | ECRG4 Augurin Precursor | 1.22 | 1.0E-02 |
| Pter | Phosphotriesterase Related | 1.20 | 1.1E-09 |
| Gpihbp1 | Glycosylphosphatidylinositol Anchored High Density Lipoprotein Binding Protein 1 | 1.17 | 5.2E-06 |
| Sdc1 | Syndecan 1 | 1.17 | 1.8E-03 |
| Cma1 | Chymase 1 | 1.16 | 3.3E-02 |
| Bend6 | BEN Domain Containing 6 | 1.16 | 4.9E-02 |
| Scrn1 | Secernin 1 | 1.15 | 2.3E-08 |
| Vwa1 | Von Willebrand Factor A Domain Containing 1 | 1.15 | 3.8E-05 |
| Cblc | Cbl Proto-Oncogene C | 1.15 | 1.0E-02 |
| Plekha4 | Pleckstrin Homology Domain Containing A4 | 1.14 | 2.9E-04 |
| Myl9 | Myosin light chain 9 | 1.13 | 4.8E-05 |
| Glrx | Glutaredoxin | 1.12 | 1.3E-04 |
| Itgb4 | Integrin Subunit Beta 4 | 1.10 | 1.5E-07 |
| Nek3 | NIMA Related Kinase 3 | 1.10 | 1.2E-02 |
| Inha | Inhibin Subunit Alpha | 1.07 | 1.7E-04 |
| Cryz | Crystallin Zeta | 1.06 | 5.0E-06 |
| Bdh1 | 3-Hydroxybutyrate Dehydrogenase 1 | 1.05 | 2.9E-17 |
| Hspa2 | Heat Shock Protein Family A (Hsp70) Member 2 | 1.05 | 3.2E-03 |
| Rps10 | Ribosomal Protein S10 | 1.04 | 8.5E-15 |
| Phlda3 | Pleckstrin Homology Like Domain Family A Member 3 | 1.04 | 7.0E-04 |
| Aatk | Apoptosis Associated Tyrosine Kinase | 1.03 | 3.3E-04 |
| Plac9 | Placenta Associated 9 | 1.02 | 3.5E-02 |
| Tspan11 | Tetraspanin 11 | 1.02 | 4.2E-02 |
| Ngdn | Neuroguidin | 1.02 | 1.7E-03 |
| Ckb | Creatine Kinase B | 1.02 | 2.3E-04 |
| S1pr3 | Sphingosine-1-Phosphate Receptor 3 | 1.01 | 8.4E-04 |
| Prss53 | Serine Protease 53 | -1.01 | 2.5E-02 |
| Mlh1 | MutL Homolog 1 | -1.01 | 2.0E-13 |
| Asb15 | Ankyrin Repeat And SOCS Box Containing 15 | -1.02 | 1.5E-07 |
| Cyp4f6 | Cytochrome P450 Family 4 Subfamily F Member 3 | -1.02 | 6.7E-07 |
| Retsat | Retinol Saturase | -1.02 | 6.0E-15 |
| Zfp871 | Zinc finger protein 871 | -1.02 | 1.1E-03 |
| Ccdc39 | Coiled-Coil Domain Containing 39 | -1.03 | 2.4E-02 |
| Myh7 | Myosin heavy chain 7 | -1.03 | 1.1E-10 |
| Ccdc62 | Coiled-Coil Domain Containing 62 | -1.03 | 1.3E-03 |
| Lepr | Leptin receptor | -1.04 | 4.2E-02 |
| Dqx1 | DEAQ-Box RNA Dependent ATPase 1 | -1.05 | 5.5E-03 |
| Zkscan8 | Zinc Finger With KRAB And SCAN Domains 8 | -1.06 | 7.0E-05 |
| Vash2 | Vasohibin 2 | -1.06 | 5.9E-04 |
| Ulbp1 | UL16 Binding Protein 1 | -1.07 | 1.5E-04 |
| Elmo3 | Engulfment And Cell Motility 3 | -1.07 | 2.9E-02 |
| Apold1 | Apolipoprotein L Domain Containing 1 | -1.07 | 6.1E-05 |
| Plekhn1 | Pleckstrin Homology Domain Containing N1 | -1.09 | 1.4E-02 |
| Rasa2 | RAS P21 Protein Activator 2 | -1.10 | 6.3E-05 |
| Nr4a1 | Nuclear Receptor Subfamily 4 Group A Member 1 | -1.11 | 3.9E-04 |
| Pfas | Phosphoribosylformylglycinamidine Synthase | -1.11 | 2.9E-05 |
| Ccnl1 | Cyclin L1 | -1.11 | 1.1E-05 |
| Spice1 | Spindle And Centriole Associated Protein 1 | -1.11 | 1.6E-03 |
| Gstz1 | Glutathione S-Transferase Zeta 1 | -1.12 | 1.8E-03 |
| Ttc14 | Tetratricopeptide Repeat Domain 14 | -1.12 | 4.3E-06 |
| Myoc | Myocilin | -1.13 | 2.8E-02 |
| Adam11 | ADAM Metallopeptidase Domain 11 | -1.14 | 2.5E-02 |
| Tent5c | Terminal Nucleotidyltransferase 5C | -1.15 | 4.1E-03 |
| Fgf7 | Fibroblast Growth Factor 7 | -1.17 | 3.7E-02 |
| Tspan33 | Tetraspanin 33 | -1.18 | 1.8E-02 |
| Slc7a4 | Solute Carrier Family 7 Member 4 | -1.19 | 1.8E-03 |
| Celsr1 | Cadherin EGF LAG Seven-Pass G-Type Receptor 1 | -1.19 | 2.7E-06 |
| Zdhhc4 | Zinc Finger DHHC-Type Palmitoyltransferase 4 | -1.20 | 2.1E-16 |
| Ddn | Dendrin | -1.21 | 3.9E-02 |
| Tsga10 | Testis Specific 10 | -1.22 | 4.7E-03 |
| Cyp4v2 | Cytochrome P450 Family 4 Subfamily V Member 2 | -1.22 | 2.7E-09 |
| Scn1a | Sodium Voltage-Gated Channel Alpha Subunit 1 | -1.22 | 6.5E-04 |
| Tnnt1 | Troponin T1, Slow Skeletal Type | -1.22 | 1.1E-03 |
| Egr1 | Early Growth Response 1 | -1.25 | 1.0E-08 |
| Plag1 | PLAG1 Zinc Finger | -1.26 | 1.1E-05 |
| Frem2 | FRAS1 Related Extracellular Matrix 2 | -1.27 | 2.4E-03 |
| Camk2b | Calcium/Calmodulin Dependent Protein Kinase II Beta | -1.28 | 2.0E-04 |
| Hpd | 4-Hydroxyphenylpyruvate Dioxygenase | -1.28 | 1.4E-03 |
| Agbl3 | AGBL Carboxypeptidase 3 | -1.30 | 2.1E-02 |
| Pik3ap1 | Phosphoinositide-3-Kinase Adaptor Protein 1 | -1.31 | 2.6E-08 |
| Amt | Aminomethyltransferase | -1.31 | 1.3E-08 |
| Ncr1 | Natural Cytotoxicity Triggering Receptor 1 | -1.41 | 4.5E-02 |
| Msi1 | Musashi RNA Binding Protein 1 | -1.43 | 3.5E-02 |
| Col17a1 | Collagen Type XVII Alpha 1 Chain | -1.44 | 1.9E-03 |
| Tbx21 | T-Box Transcription Factor 21 | -1.45 | 1.1E-02 |
| Art4 | ADP-Ribosyltransferase 4 | -1.45 | 4.7E-07 |
| Cyp4f1 | Cytochrome P450 Family 4 Subfamily F Member 2 | -1.45 | 1.9E-05 |
| Gstt3 | glutathione S-transferase, theta 3 | -1.49 | 9.4E-06 |
| Cds1 | CDP-Diacylglycerol Synthase 1 | -1.53 | 4.7E-07 |
| Irf6 | Interferon Regulatory Factor 6 | -1.55 | 2.9E-02 |
| Gcgr | Glucagon Receptor | -1.55 | 9.3E-08 |
| Slc18b1 | Solute Carrier Family 18 Member B1 | -1.56 | 3.4E-10 |
| Klrd1 | Killer Cell Lectin Like Receptor D1 | -1.56 | 1.9E-02 |
| Rubcnl | Rubicon Like Autophagy Enhancer | -1.58 | 1.5E-02 |
| Akr1c13 | Aldo-Keto Reductase Family 1 Member C3 | -1.59 | 3.2E-02 |
| Ass1 | Argininosuccinate Synthase 1 | -1.61 | 1.9E-09 |
| Rac3 | Rac Family Small GTPase 3 | -1.63 | 3.7E-02 |
| Zfp286a | Zinc Finger Protein 286A | -1.68 | 4.8E-02 |
| Sctr | Secretin Receptor | -1.79 | 1.1E-09 |
| Pcdh15 | Protocadherin Related 15 | -1.82 | 4.0E-02 |
| Myo16 | Myosin XVI | -1.82 | 1.5E-02 |
| Nsg2 | Neuronal Vesicle Trafficking Associated 2 | -1.90 | 1.9E-02 |
| Opcml | Opioid Binding Protein/Cell Adhesion Molecule Like | -1.96 | 8.3E-04 |
| Fbxw10 | F-Box And WD Repeat Domain Containing 10 | -2.05 | 1.2E-08 |
| Slc9a3 | Solute Carrier Family 9 Member A3 | -2.09 | 1.8E-03 |
| Nap1l5 | Nucleosome Assembly Protein 1 Like 5 | -2.13 | 8.9E-17 |
| Lrtm2 | Leucine Rich Repeats And Transmembrane Domains 2 | -2.27 | 4.4E-06 |
| Atp2b2 | ATPase Plasma Membrane Ca2+ Transporting 2 | -2.40 | 3.0E-19 |
| Arr3 | Arrestin 3 | -2.40 | 6.9E-04 |
| Cxcl13 | C-X-C Motif Chemokine Ligand 13 | -2.45 | 2.9E-08 |
| Rpl30 | Ribosomal Protein L30 | -2.49 | 7.6E-53 |
| Myo3b | Myosin IIIB | -2.49 | 2.2E-02 |
| Sgms2 | Sphingomyelin Synthase 2 | -2.60 | 9.7E-09 |
| Unc5cl | Unc-5 Family C-Terminal Like | -2.75 | 2.9E-02 |
| Misp | Mitotic Spindle Positioning | -2.90 | 1.1E-03 |
| Polq | DNA Polymerase Theta | -3.03 | 5.1E-05 |
| Adamts13 | ADAM Metallopeptidase With Thrombospondin Type 1 Motif 13 | -3.87 | 4.7E-10 |
| Gpr27 | G Protein-Coupled Receptor 27 | -4.08 | 3.7E-04 |
| Ptprh | Protein Tyrosine Phosphatase Receptor Type H | -4.23 | 1.1E-03 |
| Olr1085 | Olfactory receptor | -5.79 | 2.6E-03 |
